# Supplementary material for: Pediatric Venous Excess Ultrasound Score (P-VExUS): a novel approach to assess central venous pressure in the PICU
Source: Eur J Pediatr. 2026 Oct 16;185(11):855. doi: 10.1007/s00431-026-07515-z (PMC13675379; doi:10.1007/s00431-026-07515-z)
Supplement: Supplementary file 1 [file 431_2026_7515_MOESM1_ESM.docx]

**Pediatric Venous Excess Ultrasound Score (P-VExUS):**

**A Novel Approach to Assess Central Venous Pressure in the PICU**

**Authors:**

Fernando de Lima Carioca, MD^1^; Nayara Hillebrand Franzon, MD^1^; Lívia da Silva Krzesinski, MD^1^; Isabel de Siqueira Ferraz, MD^1^; Roberto José Negrão Nogueira^2^, MD, PhD; and Tiago Henrique de Souza, MD, PhD^1^.

**Affiliations:**

1. Pediatric Intensive Care Unit, Department of Pediatrics, Clinics Hospital of the State University of Campinas (UNICAMP), Campinas, SP, Brazil.
2. Department of Internal Medicine, School of Medical Sciences of the State, University of Campinas (UNICAMP), Campinas, SP, Brazil.

**Online Supplement**

**Summary**

[**Table S1.** Diagnostic performance (AUROC) of inferior vena cava–derived parameters for predicting central venous pressure >12 mmHg.	2](#_Toc223079089)

[**Table S2.** Diagnostic accuracy of P-VExUS models for predicting elevated central venous pressure (>12 mmHg). Values in parentheses indicate 95% confidence intervals.	2](#_Toc223079090)

[**Table S3.** Diagnostic accuracy of individual venous Doppler sites for predicting elevated central venous pressure (>12 mmHg). Values in parentheses indicate 95% confidence intervals.	3](#_Toc223079091)

[**Table S4.** Diagnostic accuracy of P-VExUS models for predicting low central venous pressure (<7 mmHg). Values in parentheses indicate 95% confidence intervals.	3](#_Toc223079092)

[**Table S5.** Diagnostic accuracy of individual venous Doppler sites for predicting low central venous pressure (<7 mmHg). Values in parentheses indicate 95% confidence intervals.	4](#_Toc223079093)

[**Figure S1.** Receiver operating characteristic (ROC) curves of P-VExUS models for predicting elevated central venous pressure (CVP > 12 mmHg)	4](#_Toc223079094)

[**Figure S2.** Receiver operating characteristic (ROC) curves for individual venous Doppler components in predicting elevated central venous pressure (CVP > 12 mmHg)..	5](#_Toc223079095)

[**Figure S3.** Receiver operating characteristic (ROC) curves for predicting low central venous pressure (CVP < 7 mmHg) using two pediatric P-VExUS models. .	6](#_Toc223079096)

[**Figure S4.** Receiver operating characteristic (ROC) curves of individual venous Doppler components for predicting low central venous pressure (CVP < 7 mmHg).	7](#_Toc223079097)

[**Figure S5.** Distribution of invasively measured central venous pressure (CVP) across P-VExUS Model 1 grades (A) and P-VExUS Model 2 scores (B).	8](#_Toc223079098)

# **Table S1.** Diagnostic performance (AUROC) of inferior vena cava–derived parameters for predicting central venous pressure >12 mmHg.

| **IVC-derived parameter** | **AUROC** | **95% CI** | **p-value** |
| --- | --- | --- | --- |
| IVC max/Ao | 0,520 | 0,383 to 0,656 | 0,817 |
| IVC min/Ao | 0,535 | 0,397 to 0,670 | 0,704 |
| IVC max/BSA | **0,682** | **0,544 to 0,800** | **0,041** |
| IVC min/BSA | 0,537 | 0,399 to 0,672 | 0,718 |
| IVCDI | 0,532 | 0,394 to 0,667 | 0,750 |
| IVCCI | 0,532 | 0,394 to 0,667 | 0,750 |
| ΔVCI | 0,532 | 0,394 to 0,667 | 0,750 |
| Mannarino et al.* | 0,586 | 0,446 to 0,716 | 0,282 |
| Kathuria et al.** | 0,518 | 0,381 to 0,654 | 0,829 |

***Abbreviations:*** *IVC max = maximum inferior vena cava diameter; IVC min = minimum inferior vena cava diameter; Ao = aorta; BSA = body surface area; IVCDI = IVC distensibility index; IVCCI = IVC collapsibility index; ΔIVC = respiratory variation in IVC diameter; AUROC = area under the receiver operating characteristic curve; 95% CI = 95% confidence interval; CVP = central venous pressure.*

***Age-dependent reference values reported in:***

** Mannarino S, Bulzomì P, Codazzi AC, et al. Inferior vena cava, abdominal aorta, and IVC-to-aorta ratio in healthy Caucasian children: Ultrasound Z-scores according to BSA and age. J Cardiol. 2019;74:388–393.
** Kathuria N, Ng L, Saul T, et al. The baseline diameter of the inferior vena cava measured by sonography increases with age in normovolemic children. J Ultrasound Med. 2015;34:1091–1096.*

# **Table S2.** Diagnostic accuracy of P-VExUS models for predicting elevated central venous pressure (>12 mmHg). Values in parentheses indicate 95% confidence intervals.

| **Parameter** | **P-VExUS Model 1** | **P-VExUS Model 2** |
| --- | --- | --- |
| AUROC | 0.74 (0.61 – 0.85) | 0.94 (0.84 – 0.98) |
| Cut-off | ≥ Grade II | ≥3 |
| Sensitivity | 45 (17 – 77) | 82 (48 – 98) |
| Specificity | 98 (88 – 100) | 91 (79 – 97) |
| +LR | 20.4 (2.7 – 157.8) | 9.2 (3.5 – 24.4) |
| -LR | 0.56 (0.3 – 1.0) | 0.2 (0.06 – 0.7) |
| +PV (%) | 83 (39.3 – 97.5) | 69 (46 – 86) |
| -PV (%) | 88 (81 – 92) | 95 (85 – 99) |

***Abbreviations:*** *P-VExUS = Pediatric Venous Excess Ultrasound Score; CVP = central venous pressure; AUROC = area under the receiver operating characteristic curve; +LR = positive likelihood ratio; -LR = negative likelihood ratio; +PV = positive predictive value; -PV = negative predictive value.*

# **Table S3.** Diagnostic accuracy of individual venous Doppler sites for predicting elevated central venous pressure (>12 mmHg). Values in parentheses indicate 95% confidence intervals.

| **Parameter** | **IVC** | **Hepatic Vein** | **Portal Vein** | **Renal Vein** |
| --- | --- | --- | --- | --- |
| AUROC | 0.55 (0.42 – 0.69) | 0.89 (0.77 – 0.96) | 0.80 (0.67 – 0.89) | 0.92 (0.82 – 0.98) |
| Cut-off | >0 | >0 | >0 | >0 |
| Sensitivity | 100 (71 – 100) | 91 (56 – 100) | 82 (48 – 98) | 91 (59 – 100) |
| Specificity | 11 (4 – 24) | 84 (70 – 93) | 69 (53 – 82) | 93 (82 – 99) |
| +LR | 1.12 (1.0 – 1.2) | 5.84 (2.9 – 11.8) | 2.63 (1.6 – 4.4) | 13.6 (4.5 – 41.3) |
| -LR | 0 | 0.11 (0.02 – 0.7) | 0.26 (0.07 – 0.9) | 0.097 (0.02 – 0.6) |
| +PV (%) | 22 (20 – 23.4) | 59 (41 – 74) | 39 (28 – 52) | 77 (52 – 91) |
| -PV (%) | 100 | 97 (85 – 100) | 94 (81 – 98) | 98 (87 – 100) |

***Abbreviations:*** *CVP = central venous pressure; AUROC = area under the receiver operating characteristic curve; +LR = positive likelihood ratio; -LR = negative likelihood ratio; +PV = positive predictive value; -PV = negative predictive value.*

# **Table S4.** Diagnostic accuracy of P-VExUS models for predicting low central venous pressure (<7 mmHg). Values in parentheses indicate 95% confidence intervals.

| **Parameter** | **P-VExUS Model 1** | **P-VExUS Model 2** |
| --- | --- | --- |
| AUROC | 0.69 (0.55–0.81) | 0.80 (0.67–0.89) |
| Cut-off | Grade 0 | ≤1 point |
| Sensitivity | 25 (9 – 49) | 75 (51 – 91) |
| Specificity | 100 (90 – 100) | 67 (49 – 81) |
| +LR | - | 2.25 (1.33 – 3.81) |
| -LR | 0.75 (0.58 – 0.97) | 0.38 (0.17 – 0.83) |
| +PV (%) | 100 | 56 (42 – 68) |
| -PV (%) | 71 (65 – 76) | 83 (68 – 91) |

***Abbreviations:*** *P-VExUS = Pediatric Venous Excess Ultrasound Score; CVP = central venous pressure; AUROC = area under the receiver operating characteristic curve; +LR = positive likelihood ratio; -LR = negative likelihood ratio; +PV = positive predictive value; -PV = negative predictive value.*

# **Table S5.** Diagnostic accuracy of individual venous Doppler sites for predicting low central venous pressure (<7 mmHg). Values in parentheses indicate 95% confidence intervals.

| **Parameter** | **IVC** | **Hepatic Vein** | **Portal Vein** | **Renal Vein** |
| --- | --- | --- | --- | --- |
| AUROC | 0.62 (0.48 – 0.75) | 0.70 (0.56 – 0.81) | 0.64 (0.50 – 0.76) | 0.68 (0.54 – 0.80) |
| Cut-off | ≤0 | ≤0 | ≤0 |  |
| Sensitivity | 25 (9 – 49) | 95 (75 – 100) | 75 (51 – 91) | 100 (83 – 100) |
| Specificity | 100 (90 – 100) | 44 (28 – 62) | 50 (33 – 67) | 36 (21 – 54) |
| +LR | - | 1.71 (1.26 – 2.33) | 1.50 (0.99 – 2.27) | 1.57 (1.22 – 2) |
| -LR | 0.75 (0.58 – 0.97) | 0.11 (0.02 – 0.79) | 0.50 (0.22 – 1.14) | - |
| +PV (%) | 100 | 49 (41 – 56) | 45 (35 – 56) | 46 (40 – 53) |
| -PV (%) | 71 (65 – 76) | 94 (70 – 99) | 78 (61 – 89) | 100 |

***Abbreviations:*** *CVP = central venous pressure; AUROC = area under the receiver operating characteristic curve; +LR = positive likelihood ratio; -LR = negative likelihood ratio; +PV = positive predictive value; -PV = negative predictive value.*

# **Figure S1. Receiver operating characteristic (ROC) curves of P-VExUS models for predicting elevated central venous pressure (CVP > 12 mmHg).** (A) P-VExUS Model 1 (categorical grading) demonstrated an AUROC of 0.74 (95% CI 0.61–0.85, p = 0.001). (B) P-VExUS Model 2 (point-based score) showed superior diagnostic accuracy, with an AUROC of 0.94 (95% CI 0.84–0.98, p < 0.001).

# **Figure S2.** **Receiver operating characteristic (ROC) curves for individual venous Doppler components in predicting elevated central venous pressure (CVP > 12 mmHg). (A) Inferior vena cava (IVC) score, AUROC = 0.55 (95% CI 0.42–0.69, *p* = 0.019); (B) hepatic vein score, AUROC = 0.89 (95% CI 0.77–0.96, *p* < 0.001); (C) portal vein score, AUROC = 0.80 (95% CI 0.67–0.89, *p* < 0.001); and (D) renal vein score, AUROC = 0.92 (95% CI 0.82–0.98, *p* < 0.001). Among individual sites, renal vein Doppler demonstrated the highest discriminative ability, followed by hepatic and portal veins, whereas the IVC score showed poor accuracy.**

**Figure S3.** Receiver operating characteristic (ROC) curves for predicting low central venous pressure (CVP < 7 mmHg) using two P-VExUS models. (A) P-VExUS model 1 (categorical grading) demonstrated an AUROC of 0.69 (95% CI 0.55–0.81, *p* < 0.001). (B) P-VExUS model 2 (point-based score) achieved higher accuracy, with an AUROC of 0.80 (95% CI 0.67–0.89, *p* < 0.001). Model 2 showed significantly better discriminative performance compared to model 1 (*p* = 0.02).

# **Figure S4. Receiver operating characteristic (ROC) curves of individual venous Doppler components for predicting low central venous pressure (CVP < 7 mmHg).** (A) Inferior vena cava (IVC) score, AUROC = 0.62 (95% CI 0.48–0.75, p = 0.012). (B) Hepatic vein score, AUROC = 0.70 (95% CI 0.56–0.81, p < 0.001). (C) Portal vein score, AUROC = 0.64 (95% CI 0.50–0.76, p = 0.033). (D) Renal vein score, AUROC = 0.68 (95% CI 0.54–0.80, p < 0.001).

# **Figure S5.** Distribution of invasively measured central venous pressure (CVP) across P-VExUS Model 1 grades (A) and P-VExUS Model 2 scores (B). Each point represents an individual patient measurement.
